# Sertoli-Leydig cell tumor in two siblings with *DICER1* syndrome: A case report and literature review

**DOI:** 10.1097/MD.0000000000020806

**Published:** 2020-07-02

**Authors:** Ying Zhang, Meng Ren, Yazhen Hong, Yanping Zhong, Xiaofeng Cong, Chen Chen, Ziling Liu, Yu Man, Lei Yang

**Affiliations:** a Department of Cancer Center; b Department of Pathology, The First Hospital of Jilin University, Changchun, Jinlin, China; c Translational Medicine Research Institute, Geneseeq Technology Inc, Toronto, Ontario, Canada.

**Keywords:** *DICER1*, genetic mutations, microRNA, multinodular goiter, Sertoli-Leydig cell tumors

## Abstract

**Rationale:**

*DICER1* syndrome is an autosomal-dominant tumor predisposition syndrome associated with numerous cancerous and noncancerous conditions. The most common sex cord-stromal tumor associated with *DICER1* syndrome is Sertoli-Leydig cell tumor of the ovary (SLCT), which is extremely unusual and accounts for < 0.5% of all ovarian neoplasms. SLCT predominantly affects adolescents and young female adults. To date, there are only a few case reports of ovarian SLCT with underlying germline *DICER1* mutations. The diagnosis and treatment of this rare malignancy remains challenging in the clinic mainly due to its rarity and varied presentation.

**Patient concerns:**

A 21-year-old Chinese girl (proband) was admitted in hospital for experiencing a lower abdominal pain and irregular vaginal bleeding for half a year. She was initially diagnosed with abdominal cavity mass prior to surgical operation. The other 20-year-old patient is the younger sister of the proband, who was diagnosed with ovarian cysts and had irregular menstruation and amenorrhea for 4 months. The elder sister underwent an uncomplicated bilateral ovarian tumor resection. Given a high degree of malignancy, comprehensive staged fertility-preserving surgery, including left adnexectomy, omentectomy, pelvic, and para-aortic lymphadenectomy, was performed. Since the other patient requested to maintain her fertility, tumor resection was only conducted in the right ovary.

**Diagnoses:**

The elder sister was diagnosed as poorly differentiated SLCT accompanied with heterologous stage IC rhabdomyosarcoma (RMS) based on its typical pathology features and molecular characteristics from immunohistochemistry (IHC) staining. The younger sister was diagnosed as poorly differentiated SLCT. Targeted next-generation sequencing (NGS) detected *DICER1* mutation in the plasma samples and postoperative tumor tissues of both patients.

**Interventions:**

Both patients underwent surgical tumor resection, followed by combination chemotherapy with bleomycin, etoposide, and cisplatin for 4 cycles.

**Outcomes:**

Patients received the above clinical interventions but eventually died from disease recurrence. The elder sister died from disease relapse after one and a half years postsurgery. The younger sister had a relapse of the disease 1 year later, but she refused the comprehensive staged surgery and died from disease relapse quickly.

**Lessons:**

Ovarian SLCT patients with *DICER1* mutations and a family history have a high degree of malignancy and are associated with a poor prognosis. With ongoing research efforts on *DICER1* mutations, genetic screening and counselling on a regular basis is recommended for predicting potential future cancer risk of individuals with *DICER1* syndrome family history.

## 1 Introduction

*DICER1* is a member of the RNase III family and plays an important role in the processing and maturation of miRNAs. Importantly, *DICER1* hotspot mutations lead to aberrant cell proliferation, deregulation of cell growth by genes regulating, and gonad genesis. *DICER1*-related diseases are inherited in an autosomal-dominant manner, about 80% of *DICER1* mutations are inherited from the family. Germline mutations of *DICER1* have been well documented in a wide range of tumors in cancer susceptibility syndrome—*DICER1* syndrome.

*DICER1* syndrome is a familial tumor susceptibility syndrome frequently accompanied with an increased risk of developing various types of rare benign and malignant cancers, including sex cord–stromal tumors, multilocular cystic nephroma and pleuropulmonary blastoma (PPB), and nontoxic multinodular goiter (MNG).^[1–5]^ Clinical manifestation of *DICER1* syndrome is generally present in children and young adults. In individuals with *DICER1* syndrome, Sertoli-Leydig cell tumor of the ovary (SLCT) is the most commonly observed sex cord–stromal tumor. Dicer1 is an RNaseIII endonuclease that cleaves precursor microRNAs into double-stranded miRNA duplex during miRNA biogenesis.^[6]^ After unwinding, mature active miRNA subsequently binds to downstream target genes and regulates their expression through transcriptional silencing or mRNA degradation.^[6]^ Somatic *DICER1* mutations, particularly those within the RNase IIIb domain, may lead to improperly cleaved 5p miRNA from precursor microRNA hairpin structures and complete loss of Dicer1 protein function.^[7,8]^ Although many carriers of germline *DICER1* mutations remain unaffected, previous studies have shown that most *DICER1* syndrome tumors have 1 allele with somatic missense *DICER1* mutations within 5 known hotspots in the RNase IIIb domain.^[9,10]^ Interestingly, 50% to 60% of SLCT occur in carriers of germline *DICER1* mutations and the majority of moderately or poorly differentiated cases contain somatic *DICER1* mutations.^[7,11,12]^ Here, we report that 2 siblings carrying germline *DICER1* mutations developed ovarian SLCT and multinodular goiter at the same time and the relevant literatures are also reviewed.

## 2 Case presentation

### 2.1 Case 1

The proband was a 21-year-old Chinese girl who had a lower abdominal pain and irregular vaginal bleeding for half a year. This patient was previously diagnosed with a nodular goiter 1 year ago before her visit. Physical examination revealed no obvious abdominal mass, acne, hirsutism, or clitoromegaly. Gynecological ultrasound imaging showed a large cystic light echo (∼20.8 × 15.6 cm) in the pelvic cavity as well as a ∼3.7 × 3.3 cm cystic light echo in the right ovary. Thyroid ultrasound indicated multiple thyroid nodules. Laboratory tests revealed elevated levels of cancer antigen 125 (CA125) at 251.90 U/mL (normal range: ≤ 35 U/mL), alpha-fetoprotein (AFP) at 17.71 ng/mL (normal range: ≤ 7 ng/mL), and thyroid hormone (TSH) at 6.750 μU/mL (normal range: 0.27–4.2 μU/mL). However, levels of testosterone, estradiol, follicle stimulating hormone (FSH), and luteinizing hormone (LH) remained in the normal range, and β-human chorionic gonadotropin was negative. Based on these tests, the patient was initially diagnosed with abdominal cavity mass before surgical operation.

**Figure 1:**
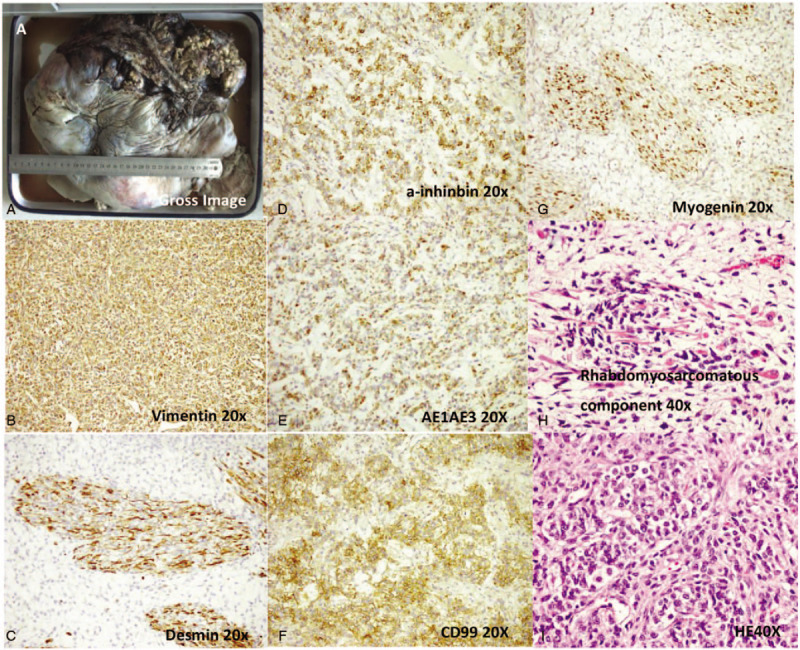
Histopathological image of the ovary with the Sertoli-Leydig cell tumor. A, The gross image of older sister: Gross examination showed its size is 37.0 × 35.0 × 13.0 cm. B–G, Positive for Vimentin, Desmin, a-inhinbin, AE1/AE3, CD99 and Myogenin expression, magnification, ×20. H, The expression of Rhabdomyosarcomatous component, magnification, ×40. I, Ematoxylin and eosin staining; magnification, ×40.

This patient underwent an uncomplicated bilateral ovarian tumor resection (size = 12 × 8 × 4.5 cm). Histopathological examination showed strings of immature and slightly atypical Sertoli cells combined with accumulation of Leydig cells. This pattern is consistent with the pathological characteristics of heterologous components in rhabdomyosarcoma (RMS). Immunohistochemistry (IHC) further revealed that tumor cells were Vimentin (+), α-Inhibin (+), CD99 (+), Calretinin (+), AE1/AE3 (+), EMA (−), PLAP (−), CD117 (−), and Ki-67 (+15%) (Table 1). She was then further diagnosed as a poorly differentiated SLCL with heterologous RMS (Fig. 1).

**Table 1:**
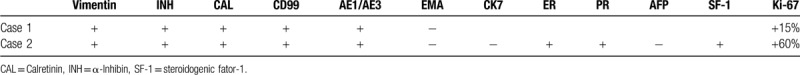
The IHC of the case 1 and case 2.

Considering a high degree of malignancy, we performed a comprehensive staged fertility-preserving surgery, including left adnexectomy, omentectomy, pelvic, and para-aortic lymphadenectomy. Postoperative histopathological examination on residual tissues from the pelvic cavity, the omentum, and lymph nodes found no evidence of tumor cells. The tumor was categorized as stage IC according to the guidelines of 2009 International Federation of Obstetrics and Gynecology. Moreover, CA125 and AFP were undetectable after tumor resection. The patient received 4 cycles of combination chemotherapy with bleomycin, etoposide, and cisplatin after surgery and was subsequently closely followed up by measuring serum CA125 level and pelvic ultrasound. Unfortunately, she died from disease relapse after one and a half years postsurgery.

### 2.2 Case 2

**Figure 2:**
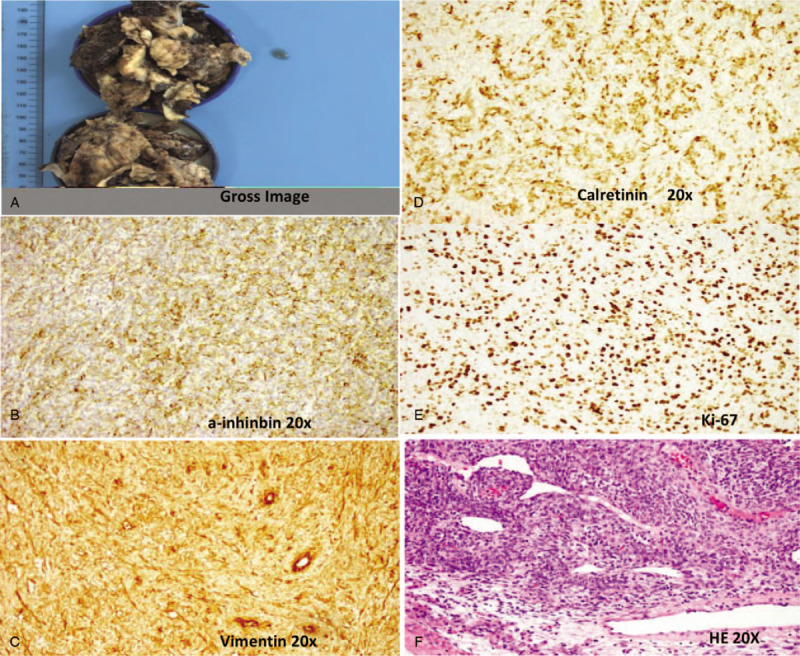
Histopathological image of the ovary with the Sertoli-Leydig cell tumor. A, The gross image of younger sister: The size of the tumor is about 12 × 8 × 4.5 cm. B–D, Imunohistochemical staining of the Sertoli-Leydig cell tumor, positive for a-inhinbin, Vimentin, Calretinin expression. Magnification, ×20. E, The expression of ki-67. F, Ematoxylin and eosin staining; magnification, ×20.

This 20-year-old patient is the younger sister of the proband. She was diagnosed with ovarian cysts a year ago and was admitted after presenting with irregular menstruation and amenorrhea for 4 months. Ultrasound and computed tomography examinations showed a complex tumor predominantly consisting of solid mass and some cystic components in the right ovary. Serum AFP level was elevated at 781.20 ng/mL, whereas serum CA-125, serum testosterone, and TSH were in their normal ranges. Since the patient requested to maintain her fertility, tumor resection was performed only in the right ovarian and no comprehensive staged surgery was performed, although bilateral ovarian biopsy samples were collected (Fig. 2). Poorly differentiated Sertoli-Leydig tumor cells were identified in the right ovarian as revealed by pathological examination. IHC analysis demonstrated that tumor cells was Vimentin (+), α-Inhibin (+), CD99 (+), Calretinin (+), SF-1(+), AE1/AE3 (+), CD10 (+), ER (+), PR (+), AFP (−), EMA (−), SMA (−), CK7 (−), Ki-67 (+60%) (Table 1). After operation, serum AFP level dropped to 105.25 ng/mL. Similar to the first patient, 4 cycles of combination chemotherapy with bleomycin, etoposide, and cisplatin was offered after tumor resection. Unfortunately, disease recurred 1 year later; the patient refused the comprehensive staged surgery option and died from disease relapse quickly.

**Figure 3:**
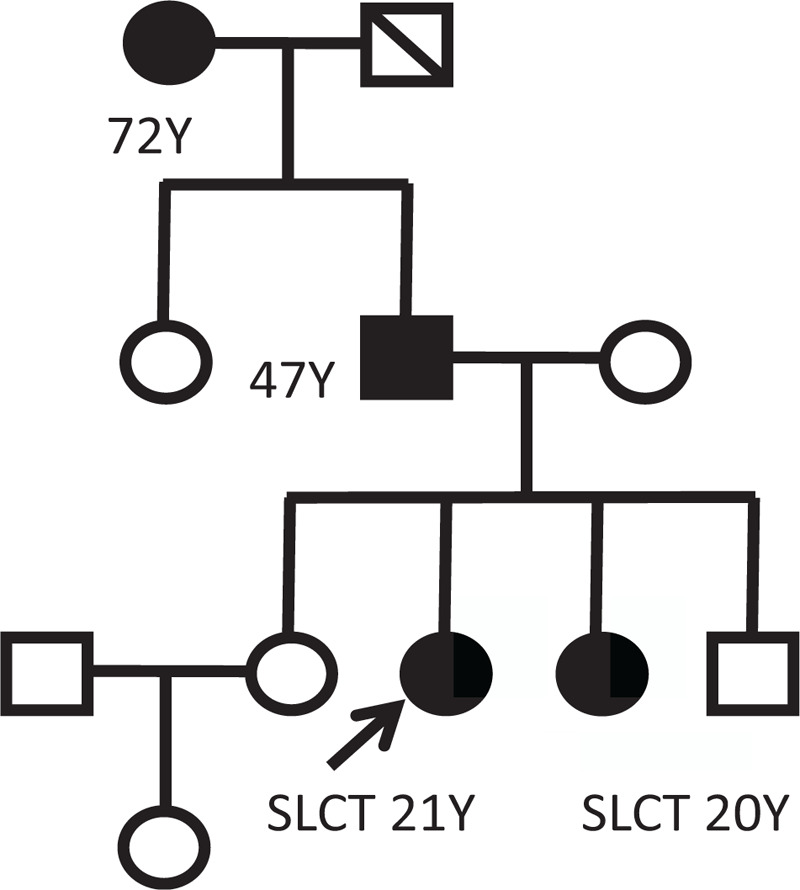
The Family map. 1. Diagonal slash indicates deceased, while the proband is indicated with an arrow. 2. Mutation-positive individuals are indicated with black. 3. MNG are indicated with +. (MNG = multinodular goiter, SLCT = Sertoli-Leydig cell tumor).

Since familial MNG and SLCT are known to predominantly be caused by *DICER1* mutations,^[5]^ we next performed a targeted NGS analysis of plasma samples and postoperative tumor tissues from both the patients. The detailed mutational profiles for *DICER1*, *FNACC*, *STAG2*, and *VEGFA* genes are summarized in Table 2. Two *DICER1* mutations were identified (c.*G5113A*, p.*E1705K*; c.*C2403A*, p.*C801X*). The abundance of the *G5113A* mutation was 21.2% and 44.3% respectively for both the patients. The elder sister also harbored a *STAG2* mutation (27.5%) and *VEGFA* gene amplification, while the younger sister contained a *FANCC* mutation (47.3%). Genetic mutation testing was subsequently carried out in their family members. Both their father and grandmother carried the same *DICER1* mutations as the siblings, and the grandmother was diagnosed with MNG rather than SLCT (Fig. 3).

**Table 2:**
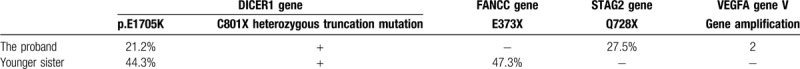
The mutation type and mutation abundance.

## 3 Discussion

SLCT is a rare type of sex cord-stromal tumor, constituting < 0.5% of all ovarian cancers. About 35% of SLCT patients have hyperandrogenism and present symptoms, such as oligomenorrhea, amenorrhea, breast atrophy, voice raucity, acne, clitoromegaly, hirsutism, and receding hairline.^[13]^ A small percentage of patients have estrogenic manifestations including menometrorrhagia and isosexual pseudoprecocity. In addition, 50% of SLCT cases without endocrine manifestations may present symptoms related to abdominal mass effect, such as abdominal discomfort or pain, ascites, and tumor rupture. These individuals can be easily misdiagnosed by a simple judgement on their abdominal symptoms.^[3]^ Gui et al^[14]^ reported that SLCT patients with no endocrine changes had poorer prognosis, indicating more aggressive biological behaviors. Consistent with this earlier study, we found that case 1 with no endocrine change had a worse clinical manifestation and a poorer prognosis compared to her younger sister who had high estrogenic manifestations.

Ovarian SLCTs can be classified into well-, moderately-, and poorly-differentiated, heterogeneous and reticular tumors according to the degree and characteristics of differentiation in the 2014 World Health Organization (WHO) guideline.^[15]^ It is increasingly accepted that the degree of differentiation of SLCT is significantly correlated with patient prognosis: highly differentiated ovarian SLCTs are usually benign, while moderately and poorly differentiated tumors containing reticular or heterogeneous components demonstrate more malignant phenotypes with poor prognosis.^[16]^

The diagnosis of SLCT mainly relies on its typical pathology features and molecular characteristics from IHC staining. The latter is commonly employed to distinguish SLCT from other ovarian malignancies by using biomarkers such as Calretinin, CD99, Inhibin, and EMA. Patients with poorly-differentiated SLCTs tend to have highly elevated AFP levels, and heterologous components may be present at least in some of poorly-differentiated tumors. Indeed, both the patients in our report had extremely high AFP levels and interstitial heterogeneous components were detected in the tumor specimen of case 1. Pathological examination confirmed that they both had poorly differentiated SLCTs.

Ovarian SLCTs have been shown to be associated with *FOXL2* and *DICER1* mutations.^[11,17,18]^*DICER1* mutations have been detected in 60% of patients with ovarian SLCTs.^[7,11,12]^ Compared with those without *DICE1* mutations, patients harboring activating *DICER1* mutations present characteristics relevant to androgenic effect, early onset, and frequent recurrence.^[19,20]^ Earlier studies showed that 80% of ovarian SLCTs had a p.E1705K *DICER1* hotspot mutation,^[4,18]^ which was also uncovered in both sisters in the present study.

SLCTs are mostly sporadic but a few familial cases have been reported to date. SLCT patients with a family history often have thyroid abnormalities such as goiter and thyroid adenoma. Rossing et al^[2]^ reported a 13-year-old girl diagnosed with ovarian SLCT and multinodular goiter. This patient, together with her brother, father, and grandfather, all harbored *DICER1* mutations. Likewise, the elder sister in this report had a nodular goiter in the past, and both patients, their father, and grandmother carried *DICER1* mutations. Together, these findings strongly suggest that for individuals affected with *DICER1* syndrome, a particular focus the clinician should make is family heredity. If a family member develops tumor(s) associated with *DICER1* syndrome, routine genetic screening and counselling on the rest members of the affected family is highly recommended for predicting potential future cancer risk.

Most ovarian SLCTs are endocrine-inactive tumors. The guidelines for treatment of SLCTs are as follows: for those with fertility requirements, fertility can be retained; for patients without fertility requirements, all uterus + double attachments can be applied as routine; for moderately or poorly-differentiated cases or tumors containing reticular and heterogeneous components, postoperative adjuvant chemotherapy is generally advocated. The commonly used chemotherapy regimen is bleomycin, etoposide, cisplatin, paclitaxel combined with the platinum regimen. Highly differentiated ovarian SLCTs are usually benign, while moderately and poorly differentiated tumors containing reticular or heterogeneous components demonstrate more malignant phenotypes with poor prognosis.

Prognosis of ovarian SLCTs is closely related to the clinical stage and pathological grade (the degree of tumor differentiation). As noted above, well-differentiated tumors are usually benign and are of low malignant potential, with a 5-year survival rate of 100%; moderately- and poorly-differentiated SLCTs are associated with 11% and 59% malignant potential, respectively, and their 5-year survival rate is about 80%.^[21]^

In summary, this case report highlights some clinical and molecular features of *DICER1* syndrome. Advances in the NGS technology have established a formidable basis for precise and early detection of key genetic alterations (e.g., *DICER1* mutations) mediating *DICER1* syndrome. It may provide clinicians with valuable information to deliver appropriate therapeutic invention and ultimately help reduce the incidence and mortality of this disease.

## 4 Conclusions

Ovarian SLCT patients with *DICER1* mutations and a family history have a high degree of malignancy and are associated with a poor prognosis. With ongoing research efforts on *DICER1* mutations, genetic screening and counselling on a regular basis is recommended for predicting potential future cancer risk of individuals with *DICER1* syndrome family history.

## Acknowledgments

The authors thank the reviewers for their helpful comments on this article and the patient for his participation and agreement to publication of the report.

## Author contributions

**Conceptualization:** Ying Zhang, Meng Ren, Lei Yang.

**Data curation:** Ying Zhang, Meng Ren, Yazhen Hong, Yanping Zhong.

**Resources:** Ying Zhang, Meng Ren, Yazhen Hong, Chen Chen, Xiao-Feng Cong.

**Writing – original draft:** Ying Zhang, Meng Ren, Yazhen Hong.

**Writing – review & editing:** Ying Zhang, Ziling Liu, Man Yu, Lei Yang.
